# Prevalence and Disability-Adjusted Life Year Rates of Asthma in China: Findings from the GBD Study 2019 of the G20

**DOI:** 10.3390/ijerph192214663

**Published:** 2022-11-08

**Authors:** Mingtao Liu, Hui Gan, Yilu Lin, Runpei Lin, Mingshan Xue, Teng Zhang, Zhangkai J. Cheng, Baoqing Sun

**Affiliations:** 1 Department of Allergy and Clinical Immunology, Department of Laboratory, National Center for Respiratory Medicine, National Clinical Research Center for Respiratory Disease, State Key Laboratory of Respiratory Disease, Guangzhou Institute of Respiratory Health, The First Affiliated Hospital of Guangzhou Medical University, Guangzhou 510150, China; 2 School of Social Sciences, Main Campus, Universiti Sains Malaysia, Gelugor 11800, Penang, Malaysia; 3 Guangzhou Laboratory, Guangzhou 510005, China; 4 Guangzhou Eighth People’s Hospital, Guangzhou Medical University, Guangzhou 510440, China; 5 Faculty of Health Sciences, University of Macau, Taipa, Macau 510060, China

**Keywords:** asthma, prevalence, disability-adjusted life years, epidemiology, GBD 2019

## Abstract

Background: The credible materials about the burden of asthma in China when compared to other countries in the group of twenty (G20) remain unavailable. Objectives and design: Following the popular analysis strategy used in the Global Burden of Disease Study, the age-, sex-, country-specific prevalence, and disability-adjusted life years (DALYs) of asthma in China were analyzed. Meanwhile, the comparison in trends between China and other countries in the G20 was also evaluated. Results: In 2019, asthma was the 8th leading cause of the DALYs’ burden of 369 diseases in China. From 1990 to 2019, the age-standardized prevalence and DALY rates of asthma in China decreased by 14% and 51%, respectively; further, the decline rate of DALYs was much higher than the global average (−51%: −43%). It is worth noting that the overall population age-standardized DALYs rate of asthma in China was the lowest in the G20 during 2019 (102.81, 95% UI: (72.30,147.42)/100,000). Moreover, the age-standardized asthma prevalence rate peaks in both childhood (178.14, 95% UI: (90.50, 329.01)/100,000) and the elderly (541.80, 95% UI: (397.79, 679.92)/100,000). Moreover, throughout the study, subjects in the 5 to 9 years old interval were a constant focus of our attention. Conclusions: The disease burden of asthma has varied greatly by gender and age over the past 30 years. In contrast to the increasing burden in most other G20 countries, the age-standardized prevalence rate of asthma shows a significant decreasing trend in China, however, the age-standardized DALYs rate shows a fluctuating change, and has even shown a rebound trend in recent years.

## 1. Background

Asthma is a common chronic airway inflammatory disease with variable prevalence and disability-adjusted life years (DALYs) over time. Moreover, asthma has been recognized as a significant public health concern since the 1970s, endangering the lives and health of people around the world in the long term [1]. In 2019, approximately 262 million people worldwide were affected by asthma and about 460,000 people lost their lives [2]. This negative effect is reported to be most common in children and young adults, mainly due to the fact that one in three individuals develop asthma [3]. Additionally, in 2019, asthma ranked 8th out of 369 diseases and injuries affecting those aged 20 and younger, as measured by DALYs [4]. Asthma has caused a sizeable economic burden to countries and regions around the world. Through the research of Australian scholars, it has been shown that the annual medical costs per person due to asthma reached thousands of dollars. In addition, the economic losses caused by unemployment and sequelae due to asthma are in the tens of billions of dollars [5]. Moreover, for individuals with severe asthma it is even more backbreaking than those without to ensure a higher quality of daily life.

The group of twenty (G20) is an international economic cooperation forum composed of developed and developing countries. Similar to other people in other countries around the world, the burden on the health of the people of these G20 countries are inextricably connected to their specific economic environments, as well as to their medical and health conditions [6,7]. However, no recent study has reported on the G20 epidemiology of asthma alone, nor has this been illustrated using the latest Global Burden of Disease (GBD) study of the G20 [1]. To date, the global burden of chronic respiratory disease has been reported in 2017 and 2019 using data from the GBD, but there have been no recent studies that have focused solely on the G20 burden of asthma [2,8]. The Global Initiative for Asthma shows that rates vary between countries, with prevalence between 1 and 18%. Further, it is more common in developed countries than in developing countries [9], however, though this is the case there are still no studies of asthma among countries within the G20. The most recent research on the status and trends of the G20 in the GBD 2019 study helps to provide direction for the development of clinical care guidelines and public health efforts; in addition, it aids policy-makers to allocate health care resources accurately and efficiently, along with reducing the social and individual burden of asthma [10]. Hence, this study aims to report the prevalence and disability-adjusted life-years (DALYs) of asthma in China and to compare these rates to other G20 countries from the period of 1990 to 2019, while utilizing data from the GBD 2019 study.

## 2. Methods

### 2.1. Data Sources

A detailed epidemiological description of the overview and definition of the GBD 2019 project can be found in the GBD 2019 Diseases and Injuries Collaborators (2020). Simply put, the GBD 2019 study used a high-ranking method in order to comprehensively assess the existing burden of diseases according to age intervals and sex groups (with both boys and males, as well as girls and females) on a global scale in 204 countries and regions spanning the years from 1990 through 2019. Next, the current research made use of the GBD 2019 methodology and specific asthma methodology for the purposes of systematic analysis of all the available demographics and epidemiology materials in China of the G20 data [2,11,12].

For the GBD 2019 study, DisMod-MR 2.1, a Bayesian meta-regression tool, was used as a primary method of estimation for disease burden. Further, by using this tool it was ensured that prevalence, DALYs due to asthma, and their uncertainty intervals were all gathered from the GBD data sources [13]. Analysts used a Gaussian, log-Gaussian, Laplace, or log-Laplace likelihood function in the DisMod-MR 2.1 model (https://ghdx.healthdata.org/gbd-2019, accessed on 26 June 2022). Moreover, all GBD asthma data used in this study were obtained from the Global Burden of Disease Collaborative Network and were filtered by control criteria—i.e., the International Classification of Diseases, Ninth Revision (code 493) and Tenth Revision (codes J45 and J46) were used to represent asthma, as well as the inclusion of the four sequelae (severity levels) [4]. The GBD estimation process is based on identifying multiple relevant data sources for each disease or injury, including censuses, household surveys, civil registration and vital statistics, disease registries, health service use, air pollution monitors, satellite imaging, disease notifications, and other sources. Each of these types of data are identified from a systematic review of published studies; searches of government and international organization websites; published reports; primary data sources such as the Demographic and Health Surveys; and the contributions of datasets by GBD collaborators.

### 2.2. Measures of Burden

Measurements of disease burden in the G20 members included prevalence and DALYs due to asthma as per the previous depiction. In addition, DALYs were calculated as a summation of years lived with disability and years of life lost. Years lived with disability is a measurement of disease burden that represents the years lived with asthma; it takes into account the duration of illness and associated disability weights so as to reflect the potential severity of the asthma in question. Years of life lost represents the number of years lost due to premature mortality through asthma. Both years lived with disability and years of life lost were evaluated for each age interval, sex group, and country in a particular year. All measurements reported were the raw values, rates per 100,000, and age-standardized rates per 100,000 as compiled from the World Health Organization’s Standard Age Structure of the World Population report. In order to calculate the age-standardized rate (ASR), which is standardized to the specific year population in terms of both prevalence and DALYs, we must first calculate the age-specific rate for each age group in a selected dataset by dividing the number of deaths by the respective population, and then multiplying the resulting number by 100,000. We must then multiply each of the age-specific rates by the proportion of the specific year population belonging to the particular age group (called the standard population weight). The age-standardized rate is obtained by adding the resulting numbers. The more detailed information about the age-standardized rate that was used can be found at http://ghdx.healthdata.org/gbd-results-tool (accessed on 26 June 2022).

### 2.3. Statistical Analysis

The quantity of prevalence and DALYs in our analysis mainly covers the number, rate, and percentage. Based on the World Health Organization’s (WHO) argument for the average age structure of the world population spanning from 2000 to 2025, We calculated age-standardized rates of prevalence and DALYs. Moreover, the prevalence and DALY rates were shown in mean rate and 95% uncertainty intervals (UIs). In this stage, the uncertainty is estimated by running each model until converged, then taking the 95th and 25th ordered samples from the 1000 posterior models as the 95% uncertainty (UI) of the estimate for each point certainty. Data were analyzed and graphical plots were produced employing Microsoft Office Excel 2022 and Prism version 9.1.1 (223) (GraphPad: San Diego, CA, USA).

To gain a better understanding of the latest trends and interpretable factors about prevalence and DALYs that are utilized in this article, we investigated the prevalence and DALYs of asthma in China in 1990 and 2019. Moreover, we made a detailed comparison of the age-standardized prevalence and DALY rates of asthma among the G20 countries (the Group of 20 countries was detailed above, however due to the lack of data from the EU as a whole and the need to incorporate data from the 28 EU members separately, a total of 43 countries were included), within the context of the global average trends between 1990 and 2019. Moreover, UIs were used to compare the prevalence and DALY rates by country and gender. Differences were considered statistically significant at the alpha level of 0.05 if the 95% uncertainty intervals of the estimates did not overlap. Subsequently, the present study aimed to assess the prevalence and DALY rates of clinically diagnosed asthma according to age and sex in China between 1990 and 2019. In regard to the GBD data management and analysis, the subjects tested were divided into the following 20 age intervals (please note there is a nearly 5 year interval in each age interval): 1 to 4, 5 to 9, 10 to 14, 15 to 19, 20 to 24, 25 to 29, 30 to 34, 35 to 39, 40 to 44, 45 to 49, 50 to 54, 55 to 59, 60 to 64, 65 to 69, 70 to 74, 75 to 79, 80 to 84, 85 to 89, 90 to 94, and 95 plus. Additionally, via the decomposition of analysis, we were able to initially explore the potential factors affecting the G20 asthma, such as population size, age and sex structure, epidemiological changes, and even asthma classification.

## 3. Results

In 2019, the prevalence cases of asthma were 24.77 (95% uncertainty interval [UI]: 20.08 to 30.67) million; further, DALYs caused by asthma were 1.41 (95% uncertainty interval [UI]: 1.05 to 1.93) million. Fortunately, death cases due to asthma were 24,750 (95% uncertainty interval [UI]: 20,244 to 30,769), which is far lower than the 40,414 cases (95% uncertainty interval [UI]: 30,325 to 58,426) in 1990. Notably, the percentage of DALYs caused by asthma was 0.37% (95% uncertainty interval [UI]: 0.29% to 0.48%), making asthma the eighth burden of 369 diseases in China in 2019 [4].

We compared the age-standardized prevalence and DALY rates of asthma in China with the global average, as well as the average of the other 42 members of the G20 countries. In 2019, the age-standardized prevalence and DALY rates in this study were far lower than the global average level [1974.16/100,000 (95% [UI]: 1530.16 to 2565.37) vs. 3415.53/100,000 (95% [UI]: 2898.92 to 4066.20); 102.81/100,000 (95% [UI]: 72.30 to 147.42) vs. 273.63/100,000 (95% CI: 216.71 to 343.38)] (Table 1).

**Figure 1:**
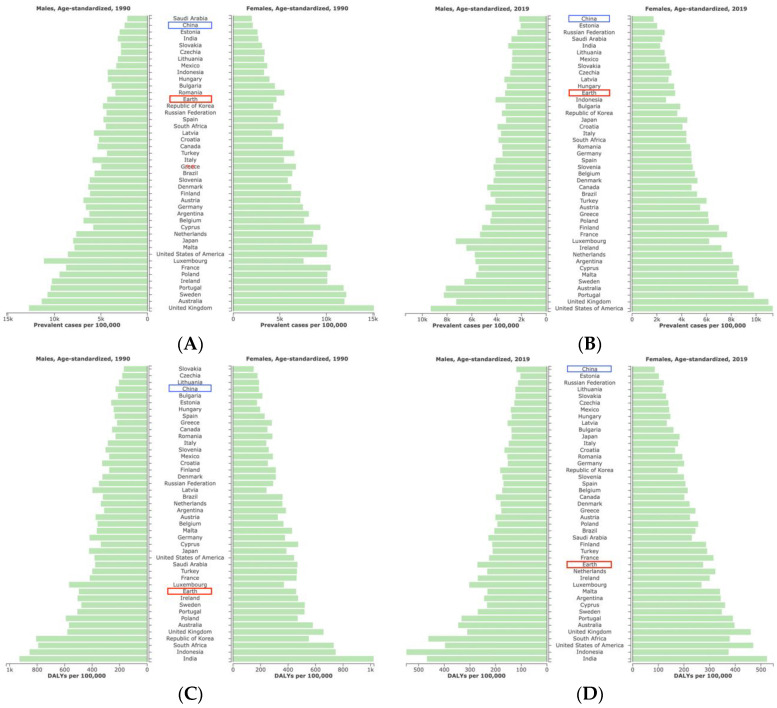
(**A**) Male age-standardized prevalent cases (per 100,000) in relation to the G20 and global average levels with asthma in 1990. (**B**) Female age-standardized prevalent cases (per 100,000) in relation to the G20 and global average levels with asthma in 2019. (**C**) Male age-standardized DALYs cases (per 100,000) in relation to the G20 and global average levels with asthma in 1990. (**D**) Female age-standardized DALYs cases (per 100,000) in relation to the G20 and global average levels with asthma in 2019. (Generated from data available at http://ghdx.healthdata.org/gbd-results-tool, accessed on 26 June 2022). The Earth entry in the graphs above represents the global average.

**Figure 2:**
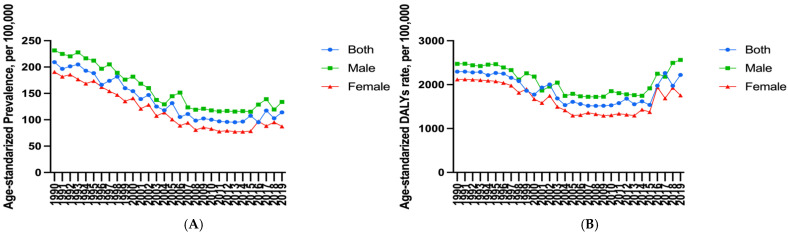
(**A**) Age-standardized prevalence rate (per 100,000) of both males and females with asthma in China during 1990 to 2019. (**B**) Age-standardized DALYs rate (per 100,000) of both males and females with asthma in China during 1990 to 2019. (Generated from data available at http://ghdx.healthdata.org/gbd-results-tool, accessed on 26 June 2022).

**Figure 3:**
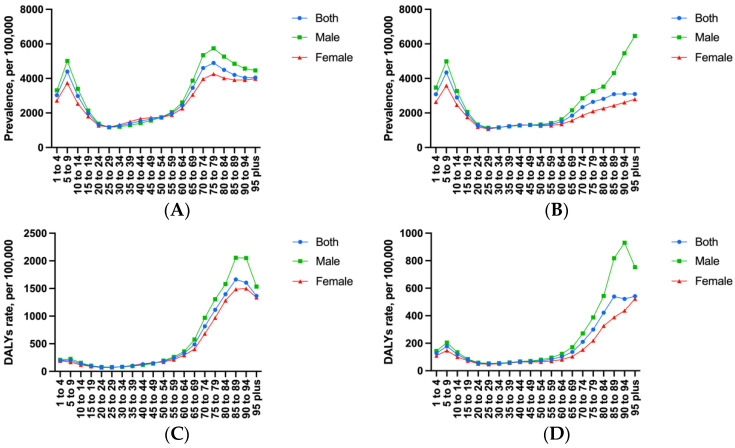
(**A**) The asthma prevalence rate (per 100,000) of different age intervals in 1990. (**B**) The asthma prevalence rate (per 100,000) of different age intervals in 2019. (**C**) The asthma DALYs rate (per 100,000) of different age intervals in 1990. (**D**) The asthma DALYs rate (per 100,000) of different age intervals in 2019 (Generated from data available at http://ghdx.healthdata.org/gbd-results-tool, accessed on 26 June 2022).

In terms of changes in the overall population age-standardized rates, the age-standardized prevalence rate of asthma in China decreased by 14% (95% [UI], −18% to −10%) between 1990 and 2019, thereby ranking 29th among the total 43 countries within the G20. Further, this was higher than the global average level [−24%, 95% [UI]: −27% to 21%)] (Table 1). However, from the overall population age-standardized prevalence rate, in 1990, asthma in China was much lower than the global average level and other G20 countries, second only to Saudi Arabia; while in 2019, this rate reached its lowest level within this specific comparison (Figure 1A,B). Most importantly, judging from the changes in each gender’s age-standardized prevalence rate of asthma in China, there was a fluctuating decline from 1990 to 2019 (Figure 2A). The age-standardized DALYs rate of asthma in China decreased by 51% (95% [UI]: −64% to −38%); moreover, China’s descending range ranked seventh in the G20 countries, followed by the Republic of Korea, Russian Federation, Japan, Poland, Germany, and Latvia. However, it must be noted that China’s rate was higher than the global average level [−43%, (95% [UI]: −48% to −37%)]. Interestingly, the overall population age-standardized DALYs rate of asthma in China jumped from the fourth lowest in 1990 (after Slovakia, Czechia, and Lithuania) to the first highest in 2019 (Figure 1C,D). In addition, the age-standardized DALYs rate of asthma for boys and males, as well as girls and females in China from 1990 to 2019 decreased first, showed a plateau, and then increased (Figure 2B). Noteworthy, the age-standardized prevalence rate of asthma showed an increasing trend in two countries, namely the United States of America and Saudi Arabia. Moreover, the age-standardized DALYs rate of asthma in the United States of America also increased, and both rates were much higher than the global average level (Table 1). Furthermore, in the 60–64 years old and 65–69 years old intervals, there was a significant increase with age; further, this increase even reached the highest rate in the 95-plus age interval of males in 2019 (Figure 3A,B). The age-standardized DALYs rate of asthma was low in childhood and youth, increased rapidly in middle and old age, and reached a peak in 85 to 89 years old. Fortunately, compared with 1990, the age-standardized DALYs rate of asthma in 2019 showed a notable decrease on the whole (Figure 3C,D).

From the perspective of the analysis of each gender, with the increase in age, the age-standardized prevalence and DALY rates of asthma in girls and females showed a wave change in total, decreasing at first, and then increasing. Among these, the rates of both females of childbearing age (15 to 49 years old) showed low levels, but the highest was in the elderly. Meanwhile, a similar trend with age occurs in boys and males. In 2019, the age-standardized prevalence and DALY rates of asthma in boys and men were higher than those in girls and women before the 30 to 34 years old interval and after the 45 to 49 years old interval. However, they were nearly equal in the 30 to 49 years old interval, which is in contrast to 1990 when both rates were higher in females than in males in the 30 to 49 years old interval. Before and after the interval of 20 to 24 years old and 50 to 54 years old, these two rates of asthma were higher in males than in females, however, there was significant overlap and shift in the 25 to 49 years old interval (Figure 2 and Figure 3). For Figure 2 and Figure 3’s data, see Table S1.

**Figure 4:**
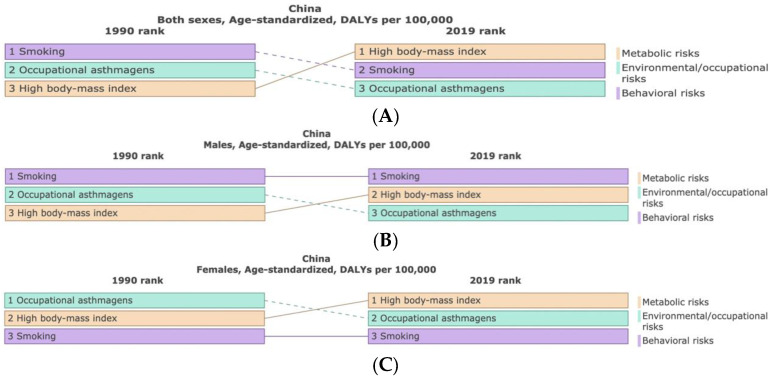
Comparison of the ranks and percentage changes in asthma that are attributable to the three highest existing risk factors data: high body mass index, smoking, and occupational asthmagens in China between 1990 and 2019. (**A**–**C**), respectively re-spent both sexes, males and females within group of comparison. (Generated from data available at http://ghdx.healthdata.org/gbd-results-tool, accessed on 26 June 2022).

Although the age-standardized DALYs rate of asthma in China can be attributed to different individual risk factors, here we will show the three main risk factors as shown by the available data: smoking, occupational asthmagens, and high body mass index, which accounted for 12.95%, 8.5%, and 5.22% of total DALYs of the overall population in 1990 (Table 2, Figure 4A), respectively. The percentage of age-standardized DALYs attributable to smoking was significantly higher in boys and males than in girls and females (23.04%: 2.11%). Conversely, occupational asthmagens are higher in girls and females, reaching 7.07%, followed by high body mass index, which was up to 5.65% (Table 2, Figure 4B,C). In 2019, the above three risk factors have reversed in the overall population, i.e., high body mass index (10.21%), smoking (10.08%), and occupational asthmagens (6.03%), respectively (Table 2, Figure 4A). Among boys and males, smoking still ranks first, but the percentage of age-standardized DALYs has decreased (23.04%: 16.9%). In contrast, a high body mass index also has a higher proportion of total DALYs in this group (4.83%: 10.38%) (Table 2, Figure 4B). In regard to girls and females—except for smoking, which was still at a relatively low level—occupational asthmagens and high body mass index showed a reversal, accounting for 5.2% and 9.97% of total DALYs, respectively (Table 2, Figure 4C).

## 4. Discussion

According to the latest report from the WHO, DALYs are “disability-adjusted life years”, and are a weighted measure of years of life lost due to premature death, and years lived with disability, which has become more popular in recent years, especially in the field of public health [14]. It is also used as a health outcome index reducing a multidimensional space of mortality, prevalence, and explicit social valuations into a single metric [15]. Therefore, using this measure in our results later may better reflect the epidemic severity of the disease. Asthma is a common chronic non-infectious disease affecting 358 million people around the world. In China, it affects about 18 people per 1000 people and has a great impact on national economic and social development, children and young people are the most commonly affected [16,17]. DALYs may better assess the burden of asthma in the background of such a disease. Further, this study is the first to elucidate asthma prevalence and DALYs in China using the G20 data.

Compared with the global average level, the burden of asthma in China has reduced significantly from 1990 to 2019; however, comparisons with the rest of the G20 members are the same. The condition situation still needs further improvement given the large population base in China, though our data indicate that the burden of asthma in China has reduced during the study period. First and foremost, among chronic respiratory diseases, asthma is ranked second, after COPD, whether on the global average or in China. Secondly, China has become one of the countries where the age-standardized prevalence and DALY rates of asthma have declined remarkably from 1990 to 2019, which, in terms of these declining rates, is much higher than the global average level. Finally, the decline in these two rates may be largely due to the population of China increasing swiftly over the past 30 years. Therefore, considering the huge population base and rapidly aging population mentioned above, the burden of asthma in China will undoubtedly face a variety of great challenges in the future, for instance, the prevalence of asthma susceptibility genes, air pollution, exposure to allergens, drug abuse and other factors [18]. There seemed to be no doubt, and it is reasonable, that asthma prevention must be possible due to the fact that standardized international epidemiological studies in both children and adults have shown that some populations have very low asthma symptom prevalence rates [19]. In China, asthma is still a long-term and extensive public health problem that seriously affects people’s life and health. Furthermore, more attention needs to be paid to this extensive problem by health authorities. Current asthma treatments broadly include antihistamines, anticongestants, antileukotrienes, bronchodilators, and steroids [20,21], which, therefore, means that the burden of asthma will continue to be monitored primarily by way of prevalence and DALY rates.

It must be noted that asthma is not constant and able to vary from remission to new asthma onset across the life course of the patient. This is particularly true if one takes into account the onset of asthma in childhood [22]. Our results in this study show that the age-standardized prevalence and DALY rates in China both reach the peak before 20 years old, most often at the interval of 5 to 9 years old, and can haul to the summit again at the elderly intervals, which, as a finding, is consistent with previous studies [4]. In addition, this disease mainly occurs in childhood and adolescence, and the age-standardized prevalence rate of asthma in boys and males is twice as high as in girls and females. In contrast, females have a slightly higher rate of asthma than males and it is more common in the young than the old [20]. A recent study reported a higher remission rate of asthma in boys and males than in girls and females, despite the shift being attributed to a later incidence of asthma in girls and females [23]. Between the ages of 10 and 18 years old, the proportion of children with asthma was 39.4% in boys and 23.4% in girls [23]. On the other hand, females are more likely to develop asthma than males [21]. Thus, gender bias in childhood asthma prevalence was reversed in adolescence and for young adults. In 2019, the age-standardized prevalence rate of asthma experienced the first peak in the 5 to 9 years old interval, and then decreased with age to an age-standardized prevalence rate in the 60 years old or older maintained at a high level. Many environmental factors are associated with the development and exacerbation of asthma, including allergens, air pollution, and other environmental chemicals [23]. Several studies have shown that allergen avoidance should be used as a causal relationship between asthma and different environmental risk factors in a preventive intervention, so as to better help policymakers draw up public health and pharmacological primary prevention measures to help reduce the prevalence and DALYs of asthma [24].

Some studies in recent years have also found that asthma is also part of the development of atopic disease; in addition, a history of allergic disease as the strongest risk factor may play a key role in the development of asthma, which is more prevalent in patients with eczema or hay fever. In addition, individuals with certain types of urticaria may also experience symptoms of asthma. Therefore, there is evidence to suggest that the incidence of asthma is closely related to the incidence of allergies. In developed countries, the incidence of allergies is high, and thus the incidence of asthma is also high. Of course, the incidence of asthma is also influenced by the diagnosis of the disease. Asthma is a heterogeneous disease [25], and the diagnosis is highly subjective, as such the differences in the incidence of asthma in different countries may also be affected by different asthma diagnostic criteria.

Our study indicates that the age-standardized prevalence and DALY rates of asthma in males are higher than those in females before the 25 to 29 years old interval in 2019, and these two rates in males and females are nearly equal at 30 to 49 years old interval. It can be concluded that the age-standardized DALYs rate of males and females decreased significantly in the 25 to 29 and 20 to 24 years old intervals, respectively. However, both then increased significantly in the 80 to 84 years old intervals. This may due to the comorbidity, additional medication for other diseases, and the diagnosis affected by the psychosocial effects of aging in the older groups [26,27]. In contrast, in 1990, these two rates in females were higher than in males at 30 to 49 years old intervals. The age-standardized prevalence of asthma changes twice before and after adolescence and in the elderly, from higher in males to nearly equal and then to higher in males again.

Several studies have also found that the prevalence and DALY rates of asthma show gender differences, as evidenced by increasing clinical and animal model shreds of evidence. This is noted in particular for adolescence, menstruation, pregnancy, menopause, and in the use of oral contraceptives. Further, these may all be associated with disease outcomes in females, and even sex hormones (particularly estrogen), in being key mediators of these differences. As a consequence, gender differences in asthma are associated with estrogen-mediated inflammation and allergic sensitivity [28,29]. Additionally, some evidence indicates that the drop in asthma prevalence observed in and around the time of adolescence in males, as well as the increased numbers of remission observed in males, is strongly suggestive of a protective role derived from the male sex hormones [30].

Asthma is caused by a combination of complex and incompletely understood environmental and genetic interactions [18]. These factors influence both its severity and its responsiveness to treatment. The recently increased rates of asthma are believed to be due to changing epigenetics (i.e., heritable factors related to DNA sequences) and a changing living environment [31]. Asthma that starts before the age of 12 years old is more likely to be due to heritable influence, while onset after age 12 is more likely to be due to environmental influences [32]. We further observe that overweight or obesity-high (BMI > 25 kg/m^2^) risk factors have become ranked first in regard to developing asthma in the overall population when compared to 1990, with females more affected by high BMI than males, which may be linked to females have a better living condition, richer diets, and less physical activity due to housework in adulthood. We should also consider the impact of weight loss (lowering BMI) on asthma management as obesity has important implications for asthma treatment in terms of medication, physiological changes in the lungs, and social effects [33].

As a bad behavior that afflicts a variety of diseases, smoking is the second largest risk factor for asthma, accounting for about 10.08% for the burden of asthma in China. Among them, the smoking burden of males (16.9%) was much higher than that of females (1.73%). The burden of asthma due to smoking has decreased in China (from 12.95% to 10.08%), however further reductions in smoking rates through effective smoking prevention programs are still needed in order to help reduce the burden of asthma. As a major factor causing occupational diseases, occupational asthmagens have increased from 8.5% in 1990 to 6.03% in 2019. Owing to the supervision of national environmental departments, corresponding preventive measures have been taken in order to strengthen the health protection of the working population. Unlike smoking and high BMI, the health effects of occupational asthma asthmagens are a relatively new field that has not been fully studied due to the low record of diseases directly related to workplace exposure and the lack of workplace exposure standards. There is, therefore, a huge opportunity to use industrial and occupational health regulations in order to prevent the development of asthma [24].

## 5. Conclusions

Our study is the most comprehensive summary of asthma prevalence and DALY rates in China from 1990 to 2019. In 2019, the age-standardized DALYs rate of asthma ranks 8th among 369 diseases in China. It is a concern that the age-standardized prevalence rate of asthma shows a significant decreasing trend, however, the age-standardized DALYs rate does show a fluctuating change; moreover, it has even shown a rebound trend in recent years. These rates were statistically significant across gender groups and age intervals. Combined with the findings of this study, the intervention of risk factors such as high BMI, smoking, and occupational asthmagens will be our next step for the formulation of preventive measures and treatment direction in order to reduce the burden of asthma.

## 6. Limitations

The quality of data that has been obtained from published and unpublished studies about asthma in the G20 data, and that are available from the GBD, is not fully satisfactory, which may affect the accuracy of the burden estimate. Firstly, the model was used in order to estimate the prevalence and DALY rates of asthma by age, sex, year, and country; further, there may be differences from the criteria of past studies, leading to discrepancies in the data collected. At the same time, the lack of relevant data in China in regard to research methods will lead to the inability to obtain detailed research results. Secondly, the GBD studies lack the use of human biochemical indicators in order to further estimate the burden of different age, and gender populations, which may result in an underestimation of the biased results within the different groups. In addition, such data may lack a certain range of effectiveness. The comparison of nearly 30 years of data do not show the impact of particular events on the disease in a specific year. It must be said that our study did not cover the burden of asthma across the various provinces in China, which may affect the national representativeness of the data. Of course, in the future, if it is possible to obtain the burden of asthma statistics from the GBD study in order to, thus, obtain more detailed information in regard to China, then we will try our best to conduct this work. In summary, the GBD data on the current burden of asthma severity still needs to be supplemented.

## Figures and Tables

**Table 1:** Age-standardized prevalence, DALYs and their changes in asthma, and the 43 members of the G20 and the world average, from 1990 to 2019. DALYs: disability-adjusted life years.

Country	Prevalence Rate			DALYs Rate		
	1990 (per 100,000)	2019 (per 100,000)	Change (%)	1990 (per 100,000)	2019 (per 100,000)	Change (%)
Global	4496.9 (3913.5–5224.3)	3415.53 (2898.92–4066.20)	−0.24 (−0.27, −0.21)	476.28 (378.49–579.59)	273.63 (216.71–343.38)	−0.43 (−0.48, −0.37)
China	2296.63 (1852.10–2880.73)	1974.16 (1530.16–2565.37)	−0.14 (−0.18, −0.10)	209.24 (159.57–272.33)	102.81 (72.30–147.42)	−0.51 (−0.64, −0.38)
Argentina	7241.79 (6184.77–8404.30)	7019.77 (5893.61–8448.07)	−0.03 (−0.13, 0.07)	352.49 (252.88–482.76)	297.63 (200.47–433.35)	−0.16 (−0.25, −0.06)
Australia	11,699.48 (10,879.25–12,543.42)	8768.43 (7162.30–10821.74)	−0.25 (−0.38, −0.09)	576.54 (421.26–775.18)	373.83 (245.52–549.84)	−0.35 (−0.46, −0.23)
Brazil	6045.9 (4725.06–7807.75)	4892.43 (3757.28–6352.04)	−0.19 (−0.25, −0.13)	343.06 (253.11–477.37)	226.7 (154.22–339.01)	−0.34 (−0.41, −0.27)
Canada	5369.62 (4412.68–6548.46)	4817.27 (3892.85–5970.94)	−0.10 (−0.25, −0.02)	254.23 (178.76–361.32)	201.86 (133.08–301.00)	−0.21 (−0.32, −0.13)
India	2970.59 (2519.45–3502.25)	2680.88 (2189.55–3221.55)	−0.10 (−0.17, −0.06)	977.17 (630.47–1427.56)	498.52 (357.50–639.83)	−0.49 (−0.59, −0.38)
Indonesia	3811.87 (3282.97–4515.18)	3431.25 (2918.28–4055.37)	−0.10 (−0.13, −0.06)	797.62 (661.05–954.67)	455.62 (372.92–549.47)	−0.43 (−0.52, −0.33)
Japan	8192.5 (6971.18–9598.36)	3865.42 (3118.67–4890.82)	−0.53 (−0.58, −0.48)	401.27 (289.81–546.61)	161.15 (104.80–241.75)	−0.60 (−0.65, −0.55)
Mexico	3534.94 (2901.91–4376.13)	2765.09 (2128.83–3625.99)	−0.22 (−0.29, −0.14)	285.39 (232.44–358.25)	142.86 (100.00–206.56)	−0.50 (−0.58, −0.41)
Republic of Korea	4491.3 (3824.36–5330.33)	3608.56 (2959.47–4514.64)	−0.20 (−0.32, −0.07)	645.6 (465.16–764.32)	178.52 (126.09–251.80)	−0.72 (−0.79, −0.61)
Russian Federation	4811.50 (4055.71–5734.01)	2520.13 (2004.47–3174.17)	−0.48 (−0.53, −0.42)	312.45 (244.22–399.65)	118.41 (81.22–172.50)	−0.62 (−0.68, −0.56)
Saudi Arabia	2092.99 (1765.37–2507.05)	2642.17 (2154.91–3207.34)	0.26 (0.17, 0.40)	416.2 (308.33–573.12)	228.29 (179.74–290.52)	−0.45 (−0.63, −0.23)
South Africa	4970.22 (3355.21–6224.02)	4123.97 (2787.98–5399.18)	−0.17 (−0.25, −0.10)	756.16 (643.75–891.52)	414.35 (342.25–518.40)	−0.45 (−0.52, −0.38)
Turkey	5503.39 (4813.36–6430.52)	5102.54 (4401.62–6040.67)	−0.07 (−0.14, −0.01)	435.17 (338.41–553.26)	253.49 (182.60–347.09)	−0.42 (−0.54, −0.31)
United Kingdom	13998.59 (11939.17–16447.44)	9166.57 (7645.04–11034.51)	−0.35 (−0.38, −0.31)	623.19 (433.44–880.48)	387.04 (255.47–566.10)	−0.38 (−0.41, −0.34)
United States of America	9374.01 (7857.79–11294.15)	10399.27 (9140.29–11903.19)	0.11 (0.02, 0.21)	417.47 (287.93–591.91)	436.01 (298.10–616.08)	0.04 (−0.03, 0.13)
Continued						
*EU*						
Austria	7014.85 (5951.85–8272.91)	5220.26 (4280.70–6380.52)	−0.26 (−0.34, −0.16)	343.21 (246.73–472.69)	212.86 (138.33–318.20)	−0.38 (−0.47, −0.30)
Belgium	7261.55 (6235.65–8543.23)	4609.89 (3791.88–5606.24)	−0.37 (−0.45, −0.26)	364.92 (265.70–501.47)	196.05 (131.16–285.57)	−0.46 (−0.54, −0.38)
Bulgaria	4196.23 (3509.42–5086.58)	3612.72 (2919.20–4427.34)	−0.14 (−0.21, −0.05)	213.74 (153.77–294.39)	149.87 (96.64–220.62)	−0.30 (−0.39, −0.21)
Croatia	5327.11 (4355.52–6448.66)	4018.57 (3316.20–4929.38)	−0.25 (−0.32, −0.18)	282.08 (205.99–382.42)	166.32 (109.02–248.94)	−0.41 (−0.49, −0.34)
Cyprus	7624.90 (7624.90–9289.55)	7088.53 (5756.88–8584.47)	−0.07 (−0.15, 0.00)	406.71 (289.04–558.36)	299.36 (199.19–439.94)	−0.26 (−0.37, −0.18)
Czechia	3146.19 (2596.81–3844.58)	3053.07 (2479.86–3781.11)	−0.03 (−0.09, 0.03)	179.79 (134.39–243.02)	133.94 (88.49–197.11)	−0.26 (−0.35, −0.17)
Denmark	6288.16 (5436.02–7394.62)	4782.68 (3910.04–5846.14)	−0.24 (−0.33, −0.14)	318.11 (231.69–432.14)	201.83 (133.39–295.31)	−0.37 (−0.45, −0.28)
Estonia	2800.52 (2426.71–3210.70)	2075.20 (1663.82–2580.60)	−0.26 (−0.35, −0.17)	208.25 (166.86–258.14)	103.88 (72.29–147.07)	0.50 (−0.59, −0.41)
Finland	6716.65 (5669.76–7866.09)	6102.50 (5043.17–7355.66)	−0.09 (−0.17, −0.02)	294.12 (201.33–422.74)	250.26 (163.90–365.94)	−0.15 (−0.22, −0.08)
France	9667.56 (8521.80–11260.00)	6555.19 (5357.38–7932.96)	−0.32 (−0.40, −0.23)	442.74 (311.18–615.72)	272.36 (180.15–401.39)	−0.38 (−0.46, −0.30)
Germany	7013.11 (6073.46–8135.45)	4153.11 (3405.37–5081.31)	−0.41 (−0.49, −0.31)	391.17 (295.58–523.01)	177.20 (117.38–258.59)	−0.55 (−0.62, −0.47)
Greece	93,070.01 (92,354.97–93,955.77)	91597.00 (90,629.04–92,581.77)	−0.02 (−0.02, −0.01)	24,431.97 (21,653.73–27,567.08)	20,200.90 (17,423.26–23,369.95)	−0.17 (−0.20, −0.15)
Hungary	94,642.90 (93,910.91–95,360.41)	92,875.82 (91,878.98–93,747.40)	−0.02 (−0.03, −0.01)	37,657.38 (34,812.66–40,821.73)	24,500.11 (20,800.48–28,628.56)	−0.35 (−0.42, −0.27)
Ireland	10213.71 (8764.93–11834.99)	6860.25 (5695.04–8348.21)	−0.33 (−0.39, −0.26)	490.56 (348.50–672.35)	286.28 (190.41–424.02)	−0.17 (−0.20, −0.15)
Italy	5629.27 (4734.78–6690.80)	4027.89 (3173.84–5106.71)	−0.28 (−0.37, −0.19)	260.13 (185.04–360.52)	163.71 (103.94–245.59)	−0.37 (−0.46, −0.29)
Latvia	4930.20 (4268.50–5758.80)	3184.73 (2584.81–4052.73)	−0.35 (−0.44, −0.25)	309.35 (238.33–401.40)	144.01 (97.39–206.98)	−0.53 (−0.62, −0.45)
Lithuania	3288.95 (2775.81–3878.73)	2718.44 (2191.37–3363.57)	−0.17 (−0.27, −0.06)	197.50 (148.63–260.16)	121.04 (82.44–176.80)	−0.39 (−0.49, −0.28)
Luxembourg	9298.17 (7741.43–11642.58)	6772.13 (5585.75–8149.60)	−0.27 (−0.38, −0.20)	464.24 (332.46–649.33)	286.67 (190.60–415.18)	−0.38 (−0.47, −0.32)
Malta	9026.04 (7735.24–10612.02)	7075.87 (5876.26–8569.64)	−0.22 (−0.30, −0.12)	399.61 (276.77–564.16)	287.08 (185.95–424.18)	−0.28 (−0.37, −0.19)
Netherlands	8134.51 (6444.96–10065.64)	6941.99 (5702.57–8418.90)	−0.15 (−0.24, 0.04)	347.79 (228.53–515.46)	278.60 (178.48–410.28)	−0.20 (−0.29, −0.04)
Poland	9777.56 (8170.35–11707.33)	5412.72 (4413.97–6639.11)	−0.45 (−0.50, −0.39)	516.38 (381.61–697.27)	226.36 (149.09–333.73)	−0.56 (−0.62, −0.50)
Portugal	11,168.28 (9284.43–13524.00)	9106.18 (7499.66–11069.60)	−0.18 (−0.28, −0.06)	513.21 (356.97–726.81)	364.35 (236.94–545.41)	−0.29 (−0.38, −0.18)
Romania	4547.38 (3707.62–5545.12)	4179.67 (3402.26–5180.64)	−0.08 (−0.17, 0.03)	260.51 (190.79–354.12)	176.01 (114.22–260.57)	−0.32 (−0.44, −0.20)
Slovakia	2996.21 (2487.21–3645.75)	2914.98 (2363.25–3588.80)	−0.03 (−0.09, 0.04)	159.34 (115.97–220.82)	127.09 (83.99–186.67)	−0.20 (−0.30,−0.12)
Slovenia	5944.55 (5013.10–7137.35)	4591.92 (3730.80–5677.77)	−0.23 (−0.29, −0.16)	275.45 (191.33–384.75)	187.58 (120.42–281.13)	−0.32 (−0.39, −0.25)
Spain	4757.39 (3990.13–5809.48)	4481.25 (3636.51–5512.17)	−0.06 (−0.17, 0.11)	233.74 (166.45–329.55)	189.85 (126.03–277.08)	−0.19 (−0.29, −0.05)
Sweden	11,444.22 (9558.65–13661.75)	7586.11 (6046.56–9402.98)	−0.34 (−0.40, −0.26)	500.65 (343.54–719.42)	309.33 (197.73–464.87)	−0.38 (−0.45, −0.31)

**Table 2:** Display of asthma changes in age-standardized DALYs due to three existing risk factors in China during 1990 and 2019.

Risk Factors	Both Sexes (%)			Males (%)			Females (%)		
	1990	2019	Changes	1990	2019	Changes	1990	2019	Changes
Smoking	13.28 (7.41–18.13)	9.36 (5.17–12.77)	−29.5	20.2 (12.16–27.52)	14.85 (8.39–20.14)	−28.3	6.27 (3.33–9.01)	4.41 (2.25–6.35)	−29.6
High body mass index	10.69 (5.53–17.82)	16.4 (10.06–24.38)	53.5	8.98 (3.99–16.76)	14.29 (8.21–22.7)	59.3	12.37 (6.43–20.28)	18.37 (11.02–27.58)	48.5
Occupational asthmagens	8.89 (7.87–10.02)	8.39 (7.54–9.28)	−5.69	11.65 (10.11–13.16)	11.14 (9.8–12.57)	−4.4	5.94 (5.12–6.84)	5.76 (5.03–6.49)	−3.1

## Data Availability

The datasets analyzed during the current study are available in the IHME data (http://ghdx.healthdata.org/gbd-results-tool, accessed on 26 June 2022). The GBD 2019 data are free to access for non-commercial users and do not require any permissions to download. Public access to the GBD 2019 data is open. The raw data from the GBD 2019 can be freely downloaded without any requirement for non-commercial users.

## Supplementary Materials

The following supporting information can be downloaded at: https://www.mdpi.com/article/10.3390/ijerph192214663/s1, Table S1. The asthma prevalence and DALYs rate in China in 1990 and 2019. DALYs: disability-adjusted life years. GBD data used in manuscript: IHME-GBD_2019_DATA-Table S2. China, age-standarized, both/male/female; IHME-GBD_2019_DATA-Table S3. China, age-standarized, both; IHME-GBD_2019_Table S4. China, age-standarized, male; IHME-GBD_2019_DATA-Table S5. China, age-standarized, female.

## Abbreviations

G20	Group of twenty
GBD	Global burden of disease
ASR	Age-standardized rate
UI	Uncertainty interval
BMI	Body mass index
