# Supplementary material for: Prevalence and Disability-Adjusted Life Year Rates of Asthma in China: Findings from the GBD Study 2019 of the G20
Source: Int J Environ Res Public Health. 2022 Nov 8;19(22):14663. doi: 10.3390/ijerph192214663 (PMC9690014; doi:10.3390/ijerph192214663)
Supplement: Supplementary file 1 [file ijerph-19-14663-s001.zip › Supplementary Table S1.pdf]

Table S1. The asthma prevalence and DALYs rate in China in 1990 and 2019. DALYs: disability-adjusted life years.

Age	1990 Prevalence, per 100,000			2019 Prevalence, per 100,000			1990 DALYs Rate, per 100,000			2019 DALYs Rate, per 100,000		
	**Both**	**Male**	**Female**	**Both**	**Male**	**Female**	**Both**	**Male**	**Female**	**Both**	**Male**	**Female**
1 to 4	3030.70	3312.01	2716.60	3085.57	3469.91	2637.43	199.90	207.81	191.06	128.15	143.94	109.74
5 to 9	4396.72	5008.67	3733.67	4343.04	4991.35	3580.11	198.05	225.14	168.69	178.14	204.74	146.85
10 to 14	2980.30	3394.15	2538.74	2898.71	3268.75	2460.14	136.54	154.75	117.11	118.86	134.18	100.69
15 to 19	1973.93	2136.84	1801.94	1914.71	2055.56	1753.53	96.96	103.11	90.47	79.56	85.39	72.90
20 to 24	1325.30	1375.06	1273.24	1267.36	1332.30	1196.32	74.62	80.19	68.79	55.12	59.01	50.87
25 to 29	1176.54	1170.02	1183.42	1110.11	1143.84	1075.14	75.95	79.00	72.74	50.26	53.64	46.76
30 to 34	1257.21	1201.71	1317.69	1159.19	1164.21	1154.06	83.33	82.00	84.79	53.31	55.30	51.29
35 to 39	1392.70	1292.90	1499.58	1234.00	1221.58	1246.90	100.57	95.19	106.32	59.00	60.66	57.28
40 to 44	1533.63	1412.72	1666.94	1294.30	1279.20	1310.03	127.40	117.78	138.01	65.75	68.01	63.40
45 to 49	1641.27	1561.31	1730.61	1304.78	1307.30	1302.16	147.38	141.41	154.05	67.47	70.86	63.94
50 to 54	1744.26	1748.11	1739.92	1296.75	1329.55	1263.64	183.80	194.24	172.06	72.61	80.45	64.70
55 to 59	1974.98	2052.02	1890.10	1347.05	1419.16	1274.27	238.47	260.89	213.76	83.17	95.79	70.43
60 to 64	2443.75	2612.05	2264.94	1492.03	1628.70	1354.06	328.21	362.20	292.09	102.11	122.66	81.38
65 to 69	3456.01	3869.58	3056.03	1853.31	2154.49	1562.96	488.05	575.70	403.27	137.06	171.45	103.91
70 to 74	4604.24	5340.08	3968.07	2340.00	2850.40	1854.56	816.06	969.74	683.20	210.55	271.50	152.58
75 to 79	4893.83	5740.45	4254.24	2644.88	3254.43	2096.36	1113.74	1304.49	969.64	299.56	388.00	219.96
80 to 84	4494.96	5261.25	4013.60	2817.66	3524.13	2264.28	1397.62	1580.57	1282.69	421.89	543.73	326.46
85 to 89	4205.52	4852.54	3912.12	3089.16	4307.37	2432.89	1663.95	2055.46	1486.41	539.36	818.30	389.09
90 to 94	4037.44	4570.98	3910.29	3101.28	5454.09	2611.96	1605.01	2052.07	1498.47	522.28	930.19	437.44
95 plus	4051.64	4466.50	3985.36	3095.19	6459.32	2798.67	1366.12	1533.41	1339.39	541.81	753.04	523.19
